# Selective potentiation of alpha 1 glycine receptors by ginkgolic acid

**DOI:** 10.3389/fnmol.2015.00064

**Published:** 2015-10-29

**Authors:** Galyna Maleeva, Svetlana Buldakova, Piotr Bregestovski

**Affiliations:** ^1^Aix Marseille Université, INS UMR_S 1106Marseille, France; ^2^INSERM, UMR_S 1106Marseille, France; ^3^Department of Cytology, Bogomoletz Institute of PhysiologyKyiv, Ukraine

**Keywords:** ligand-gated channels, glycine receptor, ion currents, whole-cell recording, patch clamp, CHO cells

## Abstract

Glycine receptors (GlyRs) belong to the superfamily of pentameric *cys*-loop receptor-operated channels and are involved in numerous physiological functions, including movement, vision, and pain. In search for compounds performing subunit-specific modulation of GlyRs we studied action of ginkgolic acid, an abundant *Ginkgo biloba* product. Using patch-clamp recordings, we analyzed the effects of ginkgolic acid in concentrations from 30 nM to 25 μM on α1–α3 and α1/β, α2/β configurations of GlyR and on GABA_A_Rs expressed in cultured CHO-K1 cells and mouse neuroblastoma (N2a) cells. Ginkgolic acid caused an increase in the amplitude of currents mediated by homomeric α1 and heteromeric α1/β GlyRs and provoked a left-shift of the concentration-dependent curves for glycine. Even at high concentrations (10–25 μM) ginkgolic acid was not able to augment ionic currents mediated by α2, α2/β, and α3 GlyRs, or by GABA_A_R consisting of α1/β2/γ2 subunits. Mutation of three residues (T59A/A261G/A303S) in the α2 GlyR subunit to the corresponding ones from the α1 converted the action of ginkgolic acid to potentiation with a distinct decrease in EC_50_ for glycine, suggesting an important role for these residues in modulation by ginkgolic acid. Our results suggest that ginkgolic acid is a novel selective enhancer of α1 GlyRs.

## Introduction

Anion-selective GlyR channels provide the inhibitory drive in the vertebrate spinal cord, brainstem, retina and some other parts of central and peripheral nervous system (Malosio et al., 1991; Lynch, 2004; Betz and Laube, 2006). Together with cation-selective nicotinic acetylcholine receptors and serotonin type 3 receptors, as well as with anion-selective GABA_A_ and GABA_C_ receptors, they belong to the superfamily of pentameric *cys*-loop receptor-operated channels (Smart and Paoletti, 2012; Lynagh and Pless, 2014).

The family of GlyRs is relatively small. Molecular cloning has enabled identification of four alpha subunits (α1–α4) and one beta (β) subunit with several splice variants (Laube et al., 2002; Lynch, 2004; Oertel et al., 2007; Dutertre et al., 2012). Functional GlyRs can be either homomeric, formed from five α subunits, or heteromeric, formed from α and β subunits with still not definitively determined stoichiometry, suggesting either 3α/2β (Langosch et al., 1988, 1990; Burzomato et al., 2003; Durisic et al., 2012) or 2α/3β (Grudzinska et al., 2005; Yang et al., 2012) composition. Alpha subunits are highly homologous, with primary structures displaying 80–90% amino acid sequence identity (Lynch, 2004); however, they differ in their kinetic properties (Takahashi et al., 1992; Singer and Berger, 1999), temporal and regional expression (Malosio et al., 1991; Betz and Laube, 2006; Heinze et al., 2007; Dlugaiczyk et al., 2008; Aroeira et al., 2011) and physiological functions (Harvey et al., 2004; Villmann et al., 2009; Dutertre et al., 2012).

Due to their diverse distribution and functions, GlyRs are potential pharmacological targets for muscle relaxant, analgesic and anti-inflammatory drugs (Webb and Lynch, 2007; Zeilhofer et al., 2012), however, only a few compounds with preferable subunit specificity are known (Yang et al., 2008; Lynch, 2009; Balansa et al., 2013).

Several studies have demonstrated that ginkgolides, extracted from the leaves of the *Ginkgo biloba* tree, are specific and potent blockers of GlyR channels (Kondratskaya et al., 2002, 2004; Hawthorne et al., 2006). *Ginkgo biloba* extract contains three groups of active substances: (i) flavonoid glycosides including quercetin and rutin; (ii) terpene trilactones (ginkgolide A, B, C, J, and bilobalide) (Ude et al., 2013); and (iii) ginkgolic acids, which are predominantly contained in the nutshells and leaves (Jaggy and Koch, 1997; Fuzzati et al., 2003).

It has been shown that ginkgolide B displays subtype-selectivity, albeit weak, with about 5- and 3-fold preferences for α1 vs. α2 and α3 GlyR subunits, respectively (Kondratskaya et al., 2005). Moreover, the inhibitory ability of ginkgolide B was 5- to 100-fold higher on heteromeric than on homomeric GlyRs, i.e., incorporation of the β subunit substantially increased the antagonism of this compound (Kondratskaya et al., 2005). Similarly to that, the other terpen trialactones from *Ginkgo biloba* extract (ginkgolide A, C, and bilobalide) block GlyR channels though with weak subunit discrimination (Hawthorne et al., 2006; Lynch, 2009). Quercetin, belonging to the flavanoid group, also inhibits α1 GlyR activity (Lee et al., 2008) in a non-competitive manner, with an IC_50_ of about 45 μM (Raafat et al., 2010). This compound also inhibits GABA_A_ and GABA_C_ receptors (Kim et al., 2015), causing seizures in animal models (Nassiri-Asl et al., 2014).

**FIGURE 1:**
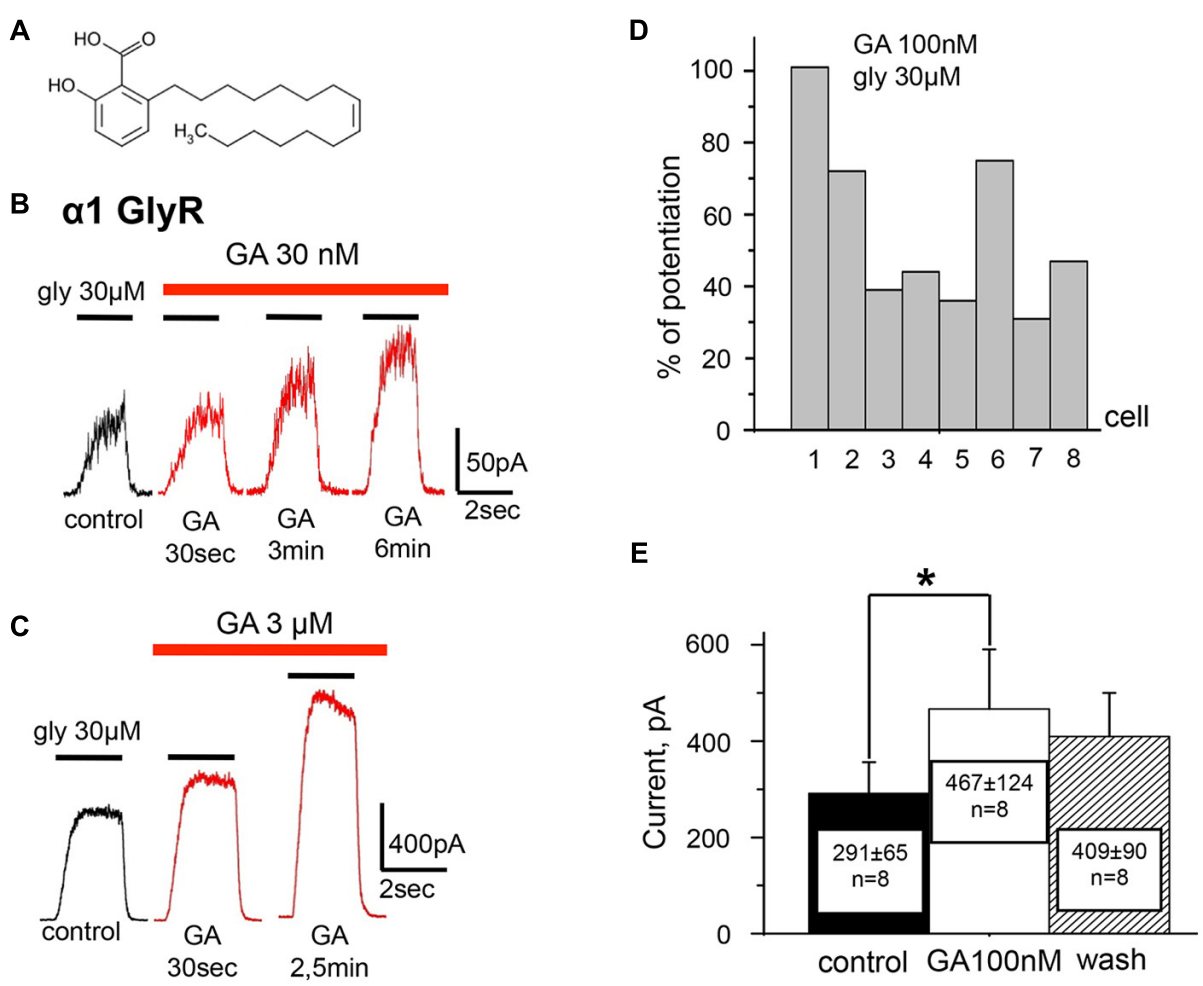
**Ginkgolic acid in nanomolar concentrations causes potentiation of I_gly_ mediated by homomeric α1 GlyRs expressed in CHO cells.** **(A)** Structure of ginkgolic acids from *G.biloba* used in this study. **(B)** Whole-cell currents induced by 30 μM glycine in control (black trace) and at the different time points of treatment of cells with 30 nM of ginkgolic acid (red traces). For this and subsequent figures, black bars above the traces indicate the time of agonist application; red bars correspond to the duration of the ginkgolic acid application. Kgluconate pipette solution, holding potential (V_h_) = 0 mV. **(C)** Examples of the whole-cell currents induced by 30 μM glycine in control (black trace) and during application of 3 μM ginkgolic acid (red traces). Note that at this concentration currents were already augmented 30 s after the beginning of ginkgolic acid application. Kgluconate pipette solution, V_h_ = 0 mV. **(D)** Percentage of potentiation by 100 nM ginkgolic acid (2 min of pre-application) of currents induced by 30 μM glycine for eight individual cells expressing α1 GlyRs. **(E)** Cumulative data on α1 GlyR-mediated current (glycine 30 μM) potentiation by 100 nM of ginkgolic acid. Mean current amplitude (pA) ± SEM from eight cells in control (black), after 2 min of ginkgolic acid (100 nM) application (white), and after washout (striped). Paired Student’s *t*-test; asterisk (^∗^) indicates significant difference, *p* < 0.05.

The functional properties of ginkgolic acid have attracted much less attention. *Ginkgo biloba* extract, used in medicine, is cleared of ginkgolic acid because of the latter’s possible side effects (Ahlemeyer et al., 2001; Hecker et al., 2002). However, it has been shown that as long as the carboxylic acid group is intact, either in free or in conjugated forms, no allergic manifestations are detected (Satyan et al., 1998). Moreover, it has been suggested that intact carboxylic acid groups are the bioactive components of the lipophilic extract of *Ginkgo biloba* leaves with antidepressant and antistress activities (Kalkunte et al., 2007). As, in contrast to ginkgolides, the effects of ginkgolic acid on the function of GlyRs and other receptor-operated channels have not been studied, we analyzed here the action of a specific compounds, a simple unsaturated (*R* = C15:1) ginkgolic acid (**Figure 1A**), on GlyRs and GABARs.

Using patch-clamp technique, we studied the effects of ginkgolic acid on ionic currents induced by activation of receptor-operated channels expressed in CHO and neuroblastoma cells. We have shown that ginkgolic acid causes specific potentiation of currents mediated by α1 GlyR subunits without strong modulation of α2, α3 GlyR, or GABA_A_ receptors. Moreover, three aminoacids, mutation of which transformed the inhibitory effect of ginkgolic acid into potentiation, were identified in α2 GlyRs.

## Materials and Methods

### Primary Culture and Transfection

The experiments were carried out on cultured Chinese hamster ovary (CHO-K1) cells obtained from the American Type Tissue Culture Collection (ATCC, Molsheim, France) and on mouse neuroblastoma cells (N2a) cells that were maintained in culture conditions as previously described (Waseem et al., 2010; Mukhtarov et al., 2013).

For electrophysiological analysis cells were transfected with cDNAs of different receptor-operated channels. One day before the transfection, cells were plated on the coverslips (12–14 mm in diameter), which were placed inside 35-mm cell culture dishes with 2 ml of medium. CHO-K1 cells were transfected with the following cDNAs encoding GlyR subunits: human α1 (1 μg/1 μl), α2 (2 μg/1 μl), α3-long (2 μg/1 μl), and β (in combination with α1 or α2 subunits with the ratio of cDNAs concentrations 1α:5β); or with a mixture of cDNAs encoding GABA_A_ receptors: α1-GFP (1 μg/μl), β2 (1 μg/μl), γ2 (1 μg/μl) using the Lipofectamine 2000 transfection protocol (Life Technology, USA). To facilitate identification of expressing cells, in the case of GlyR, green fluorescent protein (GFP, 0.5 μg/μl) was added to the transfection medium. Visualization of GABA_A_R expression was achieved by using the α1-GFP construct (Bueno et al., 1998). Three hours after the initial exposure of the cells to the cDNAs, a fresh solution replaced the old one. To prevent spontaneous activation of GlyRs by the small amount of glycine present in culture medium, strychnine (1 μM) was added to cultures expressing all types of GlyR subunits. Electrophysiological recordings were performed on fluorescent cells 24–72 h after transfection.

### Electrophysiological Recordings

Whole-cell recordings were performed at room temperature (20–25^o^C) using an EPC-9 amplifier (HEKA Elektronik, Germany). Cells were continuously superfused with external solution containing (mM): NaCl 140, CaCl_2_ 2, KCl 2.8, MgCl_2_ 4, HEPES 20, glucose 10; pH 7.4; 320–330 mOsm. Two intracellular solutions were used for filling recording patch pipettes. First, mainly used, ‘CsCl solution’ contained (mM): CsCl 140, CaCl_2_ 6, MgCl_2_ 2, MgATP 2, NaGTP 0.4, HEPES/CsOH 10, BAPTA/KOH 20; pH 7.3; 290 mOsm. In the experiments performed with CsCl intracellular solution, ionic currents were recorded at holding potential (V_h_) –30 mV. In some experiments, ‘Kgluconate solution’ was used, in which CsCl 140 mM was replaced by KCl 20 mM + Kgluconate 120 mM. Recordings with this solutions were performed at V_h_ = 0 mV. Pipettes were pulled from borosilicate glass capillaries (Harvard Apparatus Ltd, USA) and had resistances of 5–10 MOhms. For rapid replacement of solutions, the fast application system was used in this study. Two parallel rectangular tubes (100 μm × 100 μm) were positioned 40–50 μm above the recorded cell. The movement of the tubes was controlled by a computer-driven fast exchange system (SF 77A Perfusion Fast-Step, Warner, USA) allowing a 10–90% solution exchange in 3–5 ms, as measured by open electrode controls (1/10 external solution/water).

In all experiments, the duration of the pulses of agonist was 2 s. The duration of ginkgolic acid application varied from 20 s to 6 min. Cells with low input resistance (<150 MOhms) and a rapid run-down (>30% with repetitive application) were excluded from analysis.

### Data Analysis and Statistics

All electrophysiological results were analyzed using PatchMaster (HEKA Electronik, Germany) software. Dose–response curves were constructed by fitting values obtained at different concentrations, after normalization. The responses to glycine concentration were fitted using the non-linear fitting routine of the Origin 7.5 software (OriginLabs, USA) with the Hill equation:

$$I\;=\;1/\left(1\;+\;{\left(EC_{50}/\left[A\right]\right)}^{nH}\right),$$

where *I* is the normalized current amplitude induced by the agonist at concentration [A], *n*_H_ is the Hill coefficient and EC_50_ is the concentration at which a half-maximum response was induced.

Paired and unpaired Student’s *t*-tests were used for statistical analysis. The data are expressed as the means ± SEM.

### Drugs

Ginkgolic acid (C15:1, HWI Analytic GmbH, Germany) was initially dissolved in pure DMSO and then diluted with control medium to the maximal final concentration of DMSO 0.016% in experiments with using 25 μM ginkgolic acid. In test experiments, DMSO itself had no effects on the I_gly_ (data not shown; see also Mascia et al., 1996; Hall et al., 2004).

Other drugs were obtained from Tocris or Sigma–Aldrich (France).

## Results

### Low Concentrations of Ginkgolic Acid Potentiate α1 GlyRs

**FIGURE 2:**
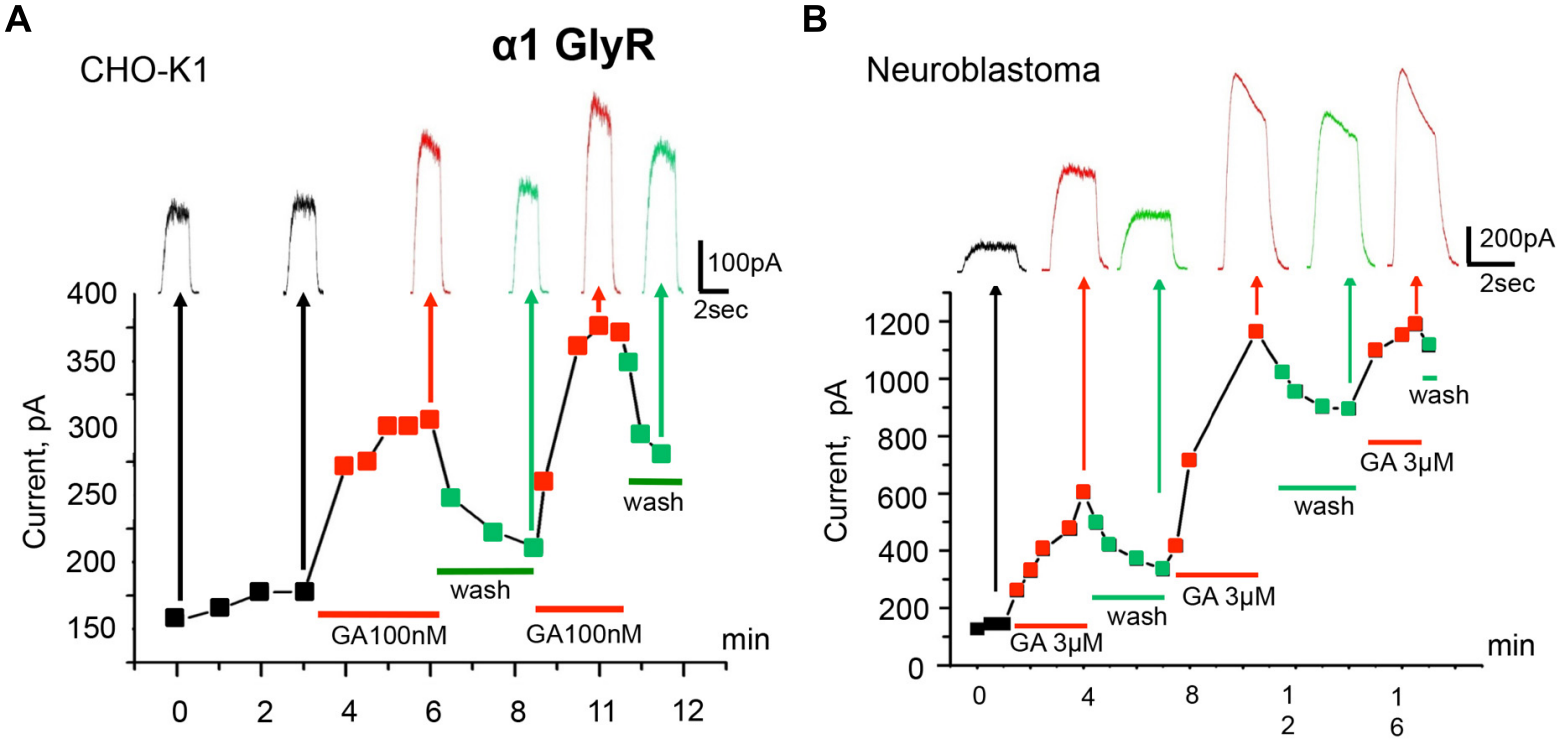
**Modulation of homomeric α1 GlyRs by ginkgolic acid.** **(A)** Time course of the development of the effect of ginkgolic acid (100 nM) on whole-cell currents induced by 30 μM glycine. Squares on the graph and traces above indicate amplitudes of the currents in control (black), in the presence of 100 nM of ginkgolic acid (red) and during washout (green). Red and green bars below the graph correspond to the duration of ginkgolic acid application and washout. Kgluconate pipette solution, V_h_ = 0 mV. **(B)** Ginkgolic acid potentiates α1 GlyRs expressed in mouse neuroblastoma (N2a) cells. Time course of I_gly_ changes during several application of the ginkgolic acid (3 μM) to N2a cells expressing α1 GlyR. Notice more than twofold increase in and partial recovery of I_gly_ after the first two applications. Kgluconate pipette solution, V_h_ = 0 mV.

To examine the effect of ginkgolic acid on the function of GlyRs, whole-cell currents in CHO cells expressing different receptor subunits were analyzed. We first investigated the effect of the acid on homomeric α1 GlyRs. To cells expressing human α1 GlyR, pulses of glycine of different concentrations and 2-s duration were applied before, during and after addition of ginkgolic acid. In contrast to the previously described inhibitory action of ginkgolides (Kondratskaya et al., 2002, 2004; Hawthorne et al., 2006), ginkgolic acid at relatively low concentrations (30 nM–3 μM) strongly enhanced whole-cell currents induced by sub-saturating (EC_10_–EC_50_) glycine concentrations (**Figures 1** and **2**). In different cells the degree of potentiation induced by pre-application of 100 nM ginkgolic acid during 2 min varied from 30 to 100% (mean = 51 ± 10%, *n* = 8; **Figure 1D**) and the average I_gly_ increased from 291 ± 65 to 467 ± 124 (*n* = 8; **Figure 1E**).

The time course of the action of 100 nM ginkgolic acid on currents induced by repetitive application of sub-saturating glycine concentration is shown in **Figure 2A**. After obtaining the whole-cell configuration and stabilization of I_gly_ amplitude (first four pulses) the external solution was changed for the one containing 100 nM of ginkgolic acid. Following the first 40 s, I_gly_ was potentiated by 54% and reached a quasi-stable level after 2 min of ginkgolic acid application (see next three pulses). Then, after washing out for 3 min and partial recovery of I_gly_ amplitude, a second application of ginkgolic acid induced an even higher and more rapidly reversible potentiation (**Figure 2A**). Similar effects were observed in neuroblastoma cells expressing α1 GlyR (**Figure 2B**) and also in outside-out patches from CHO cells expressing homomeric α1 GlyRs (potentiation from 25 to 290%, *n* = 5, data not shown).

The kinetics of the potentiation depended on the concentration of ginkgolic acid; the effect of 30 nM ginkgolic acid was observed after 2–3 min of treatment (**Figure 1B**) whereas 3 μM ginkgolic acid caused an enhancement of I_gly_ by >30% after only 30 s (**Figure 1C**). Higher concentrations of ginkgolic acid caused even more rapid enhancement of I_gly_. For instance, after 20–30 s of pre-treatment the mean potentiation induced by 25 μM ginkgolic acid was 153 ± 25% (*n* = 12), while 100 nM ginkgolic acid during the same time augmented I_gly_ by only 26 ± 8% (*n* = 8; data not shown).

**FIGURE 3:**
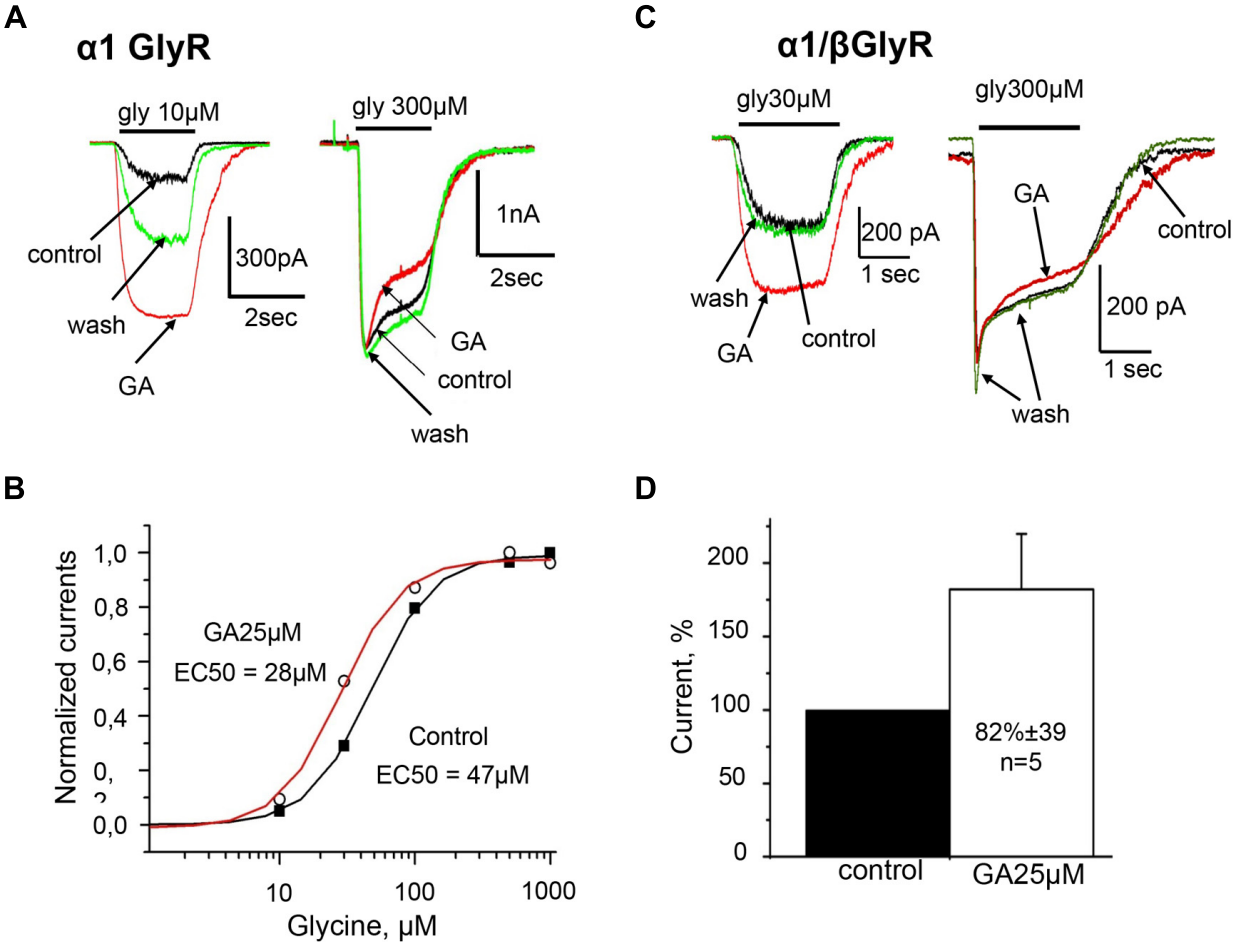
**Homomeric α1 and heteromeric α1/β GlyRs are similarly potentiated by ginkgolic acid.** **(A)** Superimposed traces of whole-cell glycine-evoked currents induced by low (10 μM; left) and high (300 μM; right) concentrations of glycine, in control (black), after ginkgolic acid application (25 μM) (red) and after washout (green). Symmetrical CsCl pipette solution, V_h_ = –30 mV. **(B)** Ginkgolic acid causes reduction in EC_50_ for glycine. Representative dose–response curve for glycine in control (black squares) and during application of 25 μM ginkgolic acid (open circles). **(C)** Superimposed traces of whole-cell glycine-evoked currents induced by low (10 μM; *left*) and high (300 μM; *right*) concentrations of glycine, in control (black), after ginkgolic acid application (25 μM) (red) and after washout (green). Recording from the cell expressing heteromeric α1/β GlyRs. Symmetrical ‘CsCl’ pipette solution, V_h_ = –30 mV. **(D)** Cumulative data. Mean percentage of α1/β GlyR-mediated current potentiation after treatment with ginkgolic acid (25 μM).

Detailed analysis at different glycine concentrations revealed that ginkgolic acid potentiated currents induced by sub-saturating doses of the agonist, while amplitudes of currents induced by saturating concentrations of glycine (0.3–1 mM) were not affected but some acceleration of desensitization kinetics was observed (**Figure 3A**). Consequently, in the presence of ginkgolic acid, dose–response curves shifted to the left. **Figure 3B** represents an example of EC_50_ shift from 47 μM in control to 28 μM after application of 25 μM ginkgolic acid. On average, 25 μM ginkgolic acid caused a significant decrease in EC_50_ for glycine from 36 ± 3 μM (*n* = 6) to 22 ± 1.4 μM (*n* = 6; *p* < 0.01). Similar shift in EC50 was observed also at using lower concentration of ginkgolic acid. For instance, pretreatment of cells with 3 μM ginkgolic acid during 1–3 min caused a significant shift (*p* < 0.01) of EC_50_ from 36 ± 6 μM in control to 17 ± 2 μM (*n* = 9) after application of the acid (data not shown).

These observations demonstrate that ginkgolic acid, albeit with slow kinetics, is capable of causing strong potentiation of α1 GlyR even in the nanomolar range of concentrations.

### Effect of Ginkgolic Acid on Heteromeric α1/β GlyRs

Some antagonists of GlyRs exhibit different abilities to change the activities of homomeric and heteromeric receptors. For instance, the plant alkaloid picrotoxin more effectively inhibits homomeric GlyR than heretomeric α/β receptors (Pribilla et al., 1992; Pistis et al., 1997), while ginkgolide B more effectively antagonizes heteromeric GlyRs (Kondratskaya et al., 2005). To clarify whether ginkgolic acid exhibits the homo/hetero subunit selectivity we studied its action on heteromeric α1/β receptors.

Similarly to homomeric α1 GlyR, whole-cell currents induced by glycine concentrations below EC_50_ (30 μM) were strongly potentiated by ginkgolic acid (**Figure 3C**, left). On average, currents induced by subsaturating glycine concentrations increased by 82 ± 39% (*n* = 5) in comparison with control (**Figure 3D**).

Similarly to homomeric α1 GlyRs, ginkgolic acid did not increase currents induced by application of saturated glycine concentrations (0.3–1 mM) to heteromeric α1/β receptors (**Figure 3C**, right).

### Effect of Ginkgolic Acid on α2 GlyRs

Before the analysis of the action of ginkgolic acid on GlyRs formed of α2 subunits, we estimated its EC_50_ by obtaining dose–response curves. The EC_50_ to glycine varied from 24 to 69 μM with a mean value 42 ± 2 μM (*n* = 10; data not shown), i.e., slightly higher than for α1 GlyRs.

**FIGURE 4:**
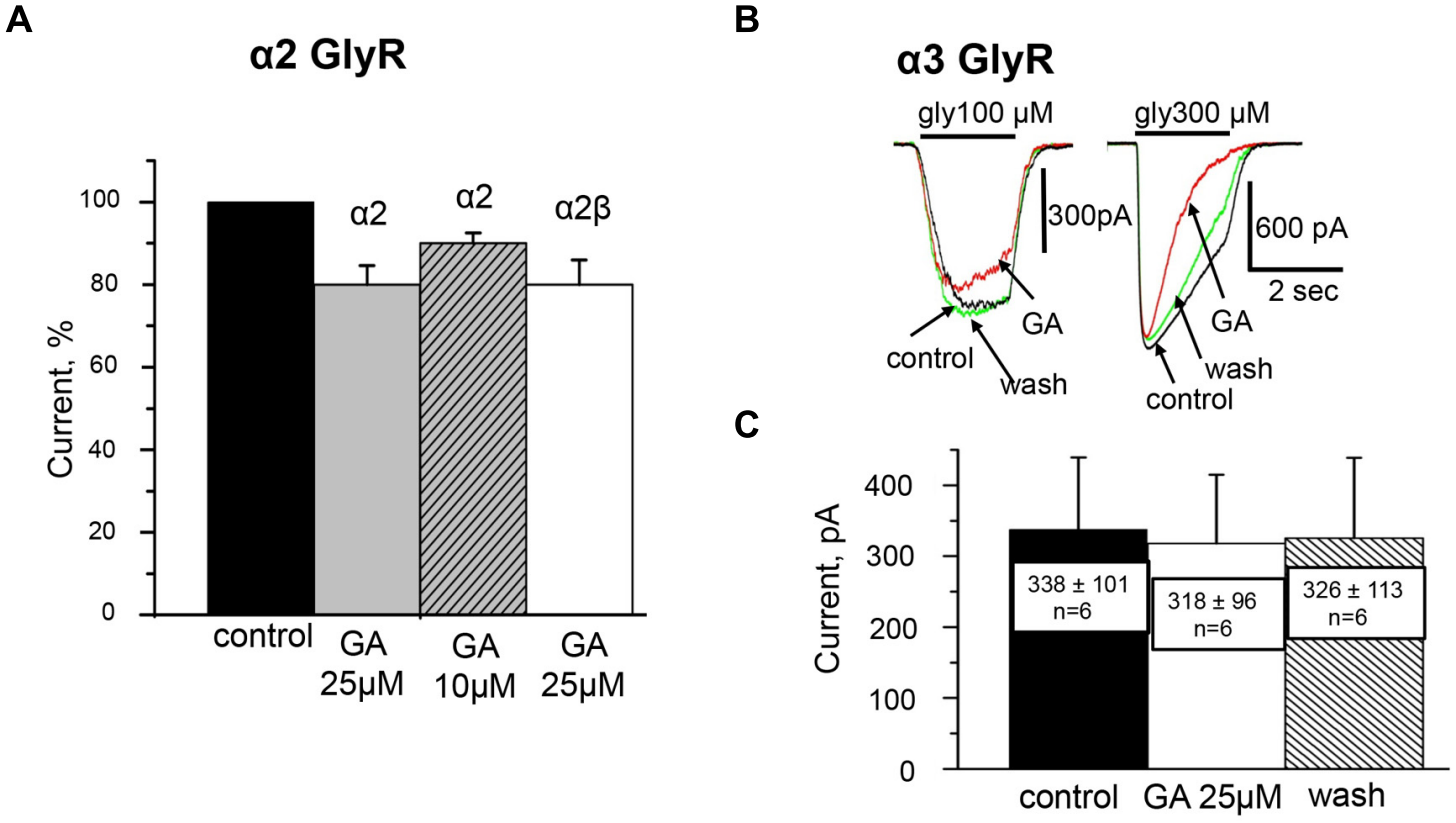
**Effect of ginkgolic acid on I_gly_ mediated by homomeric α2 and α3 GlyRs and heteromeric α2/β GlyRs.** **(A)** Cumulative data. Mean percentage of the effect of 25 μM ginkgolic acid on homomeric α2 GlyRs (light gray), heteromeric α2/β GlyRs (white colomn), and 10 μM of ginkgolic acid on α2 GlyRs (striped). Glycine 30 μM was applied. Data from 7 to 12 cells for each case. **(B)** Superimposed traces of glycine-evoked currents induced by low for this subunit (100 μM; *left*) and high (300 μM; *right*) concentrations of glycine, in control (black), after ginkgolic acid application (red) and after washout (green). Symmetrical ‘CsCl’ pipette solution, V_h_ = –30 mV. **(C)** Summary of the data on the effect of ginkgolic acid on the α3-long subunit of GlyR. Mean amplitudes of currents (pA) ± SEM induced by 100 μM glycine from six cells in control (black), during ginkgolic acid application (white), and after washout (striped).

In contrast to the action on α1 GlyRs, low concentrations of ginkgolic acid (<10 μM) had no effect on the amplitude of I_gly_. At ginkgolic acid concentrations of 10 μM or higher, a small inhibition of currents was observed. Thus, 10 and 25 μM of ginkgolic acid inhibited α2 GlyRs by about –10 ± 3% (*n* = 8) and –20 ± 5% (*n* = 11), respectively (**Figure 4A**). However, in many cells high doses of ginkgolic acid stimulated non-reversible run-down, which could be an additional reason for this small inhibition. At low concentrations (1 μM) the effect of ginkgolic acid was not detectable; with a long application (5–6 mins) even the tendency to weak elevation of I_gly_ was observed. This may result partially from a spontaneous run-up of responses during long-lasting whole cell recordings (data not shown, but see Fucile et al., 2000).

High concentrations of ginkgolic acid also caused a weak inhibition of heteromeric α2/β receptors (–14 ± 4%, *n* = 9). **Figure 4A** summarizes the action of high ginkgolic acid doses on α2 and α2β receptors.

### Effect of Ginkgolic Acid on α3 GlyRs

The human α3 GlyR subunit exists in two splice variants, α3K (short) and α3L (long), the last one bears an additional segment of 15 amino acids within the cytoplasmic TM3-TM4 loop (Breitinger et al., 2002) To analyze the action of ginkgolic acid on α3 GlyRs we selected α3L splice variant as its TM3–TM4 insert is important for spatial structure stabilization of the cytoplasmic domain and it is involved in the regulation of GlyR channel gating (Breitinger et al., 2009).

Analysis of concentration dependencies showed that for GlyRs formed of this subunit the EC_50_ to glycine in control was 142 ± 9.8 μM (*n* = 11; data not shown), i.e., about threefold higher than for α1 and α2 GlyRs.

As illustrated in **Figures 4B,C**, the effect of ginkgolic acid (25 μM) on the amplitude of whole-cell currents recorded from CHO cells expressing α3 GlyR was negligible. In more detail, after 20–40 sec of pre-treatment with ginkgolic acid, the currents induced by a concentration of glycine ‘below EC_50_’ (100 μM) slightly decreased (**Figure 4B**, left), on average by –9 ± 2% (from –4 to –11%; *n* = 4); two other cells showed no effect and in one cell a weak potentiation (+4%) was observed. For this concentration, mean currents in control, in the presence of ginkgolic acid and after washout were, respectively, 338 ± 101 pA, 318 ± 96 pA, and 326 ± 113 pA (*n* = 6; **Figure 4C**).

Similarly to its action on α1 and α2 subunits, ginkgolic acid accelerated the desensitization kinetics of currents induced by application of saturated glycine concentrations (≥300 μM; **Figure 4B**, right).

These data indicate that ginkgolic acid, even at high doses, is not capable of potentiating the function of α2 and α3 GlyRs.

### Effect of Ginkgolic Acid on GABA_A_Rs

We further analyzed the action of ginkgolic acid on GABA receptors expressed in CHO cells. Its effect was studied on the most widespread in mammalian brain GABA_A_R combination – α1/β2/γ2 (Olsen and Sieghart, 2008). We assume that all cells that demonstrated GABA-evoked currents expressed on their surface α1/β2/γ2 receptors, as it was shown before that α1γ2, β2γ2 and homomeric receptors are retained within the endoplasmic reticulum (Connolly et al., 1996; Gorrie et al., 1997). Moreover, cells transfected only with β2 subunits do not produce ionic currents (Connolly et al., 1996) or surface staining (Taylor et al., 1999).

**FIGURE 5:**
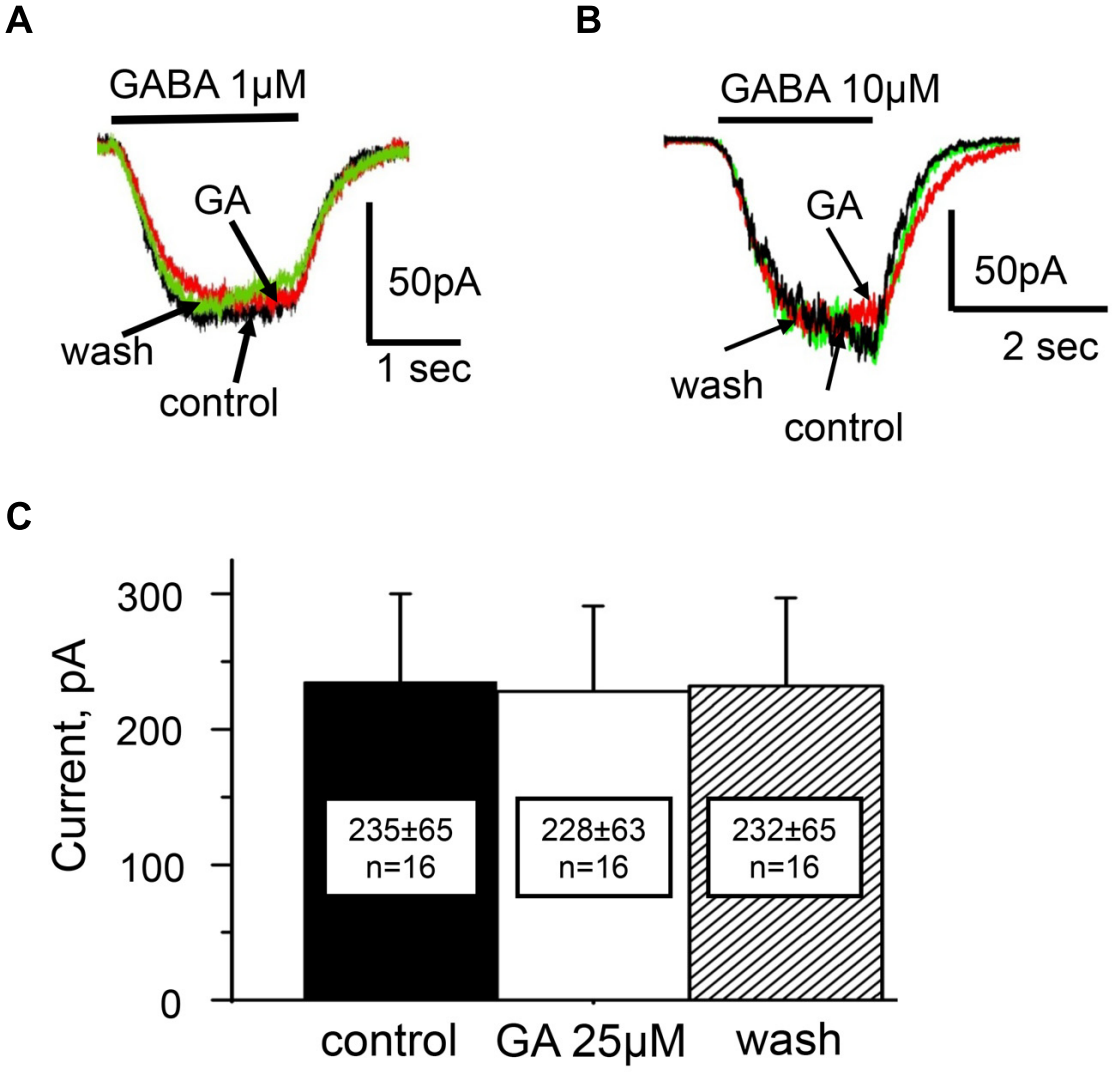
**Action of ginkgolic acid on GABA-evoked currents recorded in CHO cells, expressing of GABA_A_ receptors.** **(A,B)** Superimposed traces of currents evoked by GABA 1 μM **(A)** and 10 μM **(B)** in control (black), after ginkgolic acid application (red) and after washout (green). V_h_ = –30 mV. Symmetrical ‘CsCl’ pipette solution. **(C)** Summary of the data on the effect of ginkgolic acid on α1/β2/γ2 GABARs. Mean amplitudes of currents (pA) ± SEM induced by 10 μM GABA from 16 cells in control (black), during ginkgolic acid application (white) and after washout (striped).

Analysis of concentration dependencies revealed that the EC_50_ of GABA for GABA_A_Rs in control solution was 11 ± 1 μM (data not shown). The action of ginkgolic acid was tested on 16 cells at using concentration of GABA close to EC_50_ (10 μM) and on three cells using concentration of GABA close to EC_10_ (1 μM). After 20–40 s of treatment with ginkgolic acid (25 μM), we did not observe any changes in GABA-evoked currents, either with EC_10_ (**Figure 5A**) or with EC_50_ (**Figure 5B**) concentrations of GABA. For 10 μM GABA the mean currents in control, after ginkgolic acid application and after washout were 235 ± 65 pA, 228 ± 63 pA, and 232 ± 65 pA (*n* = 16), respectively (**Figure 5C**).

Thus, similarly to the α3 GlyR, there was no significant difference between GABA-induced currents for the α1/β2/γ2 combination of GABA_A_Rs before and after ginkgolic acid (25 μM) application.

### Amino Acids Involved in the Modulation of GlyRs by Ginkgolic Acid

Finally, we searched for the residues responsible for the different actions of ginkgolic acid on α1 and α2 GlyR subunits. Recent studies have identified several residues involved in allosteric modulation of different GlyR subunits (Xiong et al., 2011; Yevenes and Zeilhofer, 2011a). Among them are the S296 residue in the third TM3, as well as alanine 52 in the extracellular region and glycine 254 in the TM2 domain.

**FIGURE 6:**
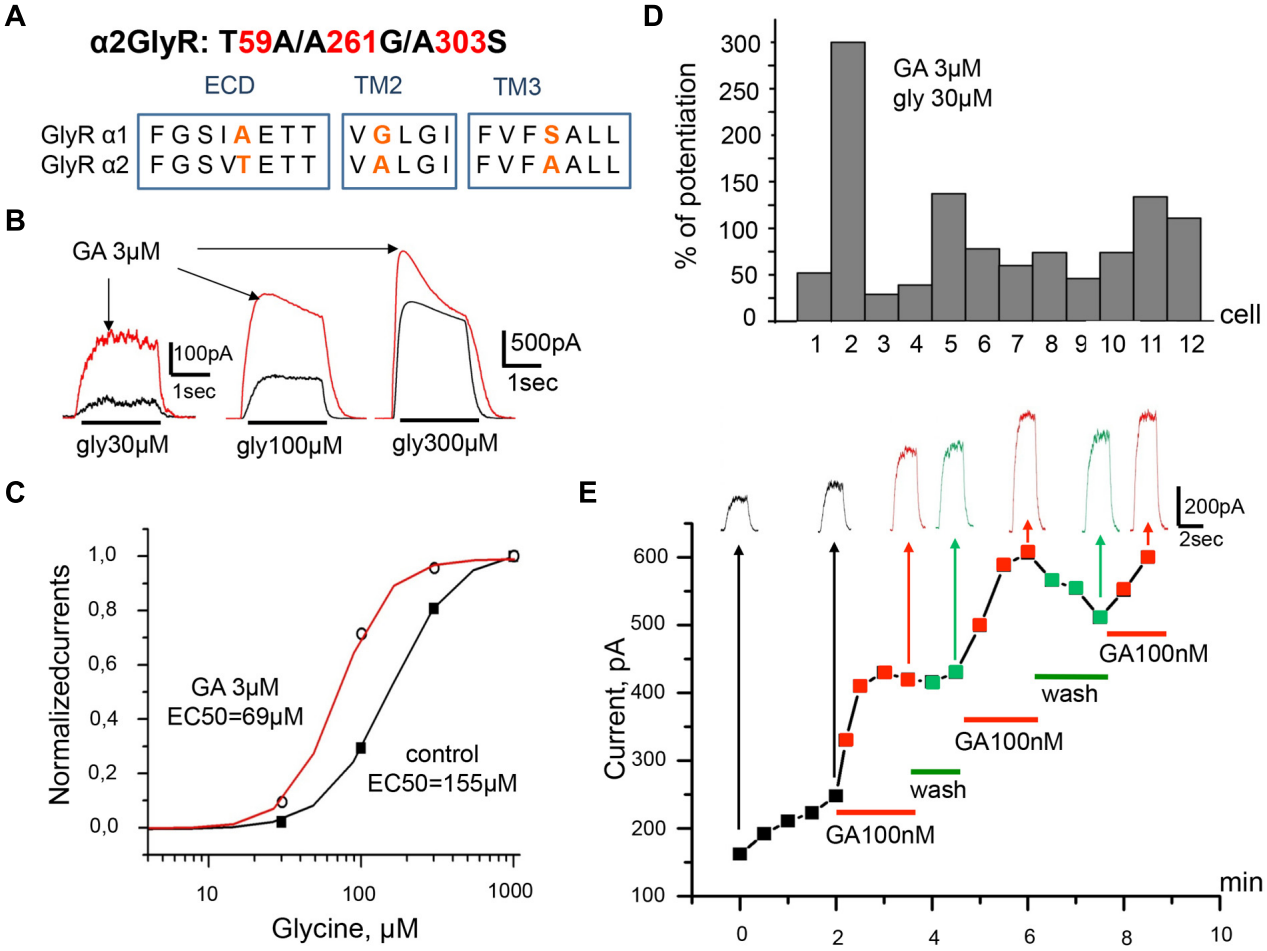
**Ginkgolic acid causes potentiation of I_gly_ on cells expressing the mutant α2 (T59A/A261G/A303S) GlyRs.** **(A)** Primary sequence alignment of α1 and α2 GlyR subunits in the extracellular loop, TM2 and TM3 domains; substituted residues are in red. **(B)** Superimposed traces of glycine-evoked currents induced by different glycine concentrations (30, 100, 300 μM) in control (black) and after ginkgolic acid (3 μM) application (red). Kgluconate pipette solution, V_h_ = 0 mV. **(C)** Ginkgolic acid causes a reduction in EC_50_ for glycine. Normalized dose–response curves for glycine in control (black squares) and during application of 3 μM of ginkgolic acid (open circles). **(D)** Percentage of potentiation of glycine (30 μM) evoked currents after ginkgolic acid (3 μM) application for eight individual cells expressing α2 (T59A/A261G/A303S) GlyRs. **(E)** Example of the time course of the development of the ginkgolic acid effect (3 μM) on the amplitude of the ionic currents mediated by α2 (T59A/A261G/A303S) GlyRs; glycine 30 μM was applied. Kgluconate pipette solution, V_h_ = 0 mV.

As some parts of the chemical organization of endocannabinoids and ginkgolic acid show high similarity we investigated whether there are also similarities of functional effects. Indeed, both compounds produce potentiation of α1 GlyR subunits. Molecular sites for allosteric control of GlyRs by the endocannabinoid have been identified (Yevenes and Zeilhofer, 2011a). It has been shown that substitution in the α2 subunit of residues T59, A261, and A303 (**Figure 6A**) for corresponding residues from the α1 subunit (A52, G254, and S296) converts the effect of N-arachidonoyl–glycine from inhibition to potentiation (Yevenes and Zeilhofer, 2011a).

In order to check whether the same amino acid residues are indispensable for positive modulation of α1 GlyR by ginkgolic acid we performed T59A/A261G/A303S substitution in α2 subunit (**Figure 6A**) and studied the effect of ginkgolic acid on currents mediated by this α2 GlyR mutant. Ginkgolic acid applied to CHO cells expressing α2 T59A/A261G/A303S subunits caused potentiation of responses to glycine, similar to those observed for α1 GlyR (**Figure 6B**). After pre-application for 1–2 mins of 3 μM ginkgolic acid, currents induced by non-saturating glycine concentrations (30 μM) increased in different cells in the wide range from 25 to 300% (**Figure 6D**), with a mean value of 95 ± 21% (*n* = 12).

Analysis of concentration dependencies revealed that the sensitivity of the mutant GlyR to glycine was weaker than that of WT α2 GlyR. In control conditions, the glycine EC_50_s for α2 mutant receptors varied from 56 to 238 μM. In the presence of 3 μM ginkgolic acid, dose–response curves showed a distinct left-shift (EC_50_ values varied from 31 to 118 μM). Thus, in the cell illustrated in **Figure 6C**, in control conditions EC_50_ was 157 μM and it became 69 μM in the presence of ginkgolic acid. On average, ginkgolic acid caused a significant (*p* < 0.05) decrease in EC_50_ from 119 ± 16 μM in control to 76 ± 9 μM (*n* = 12) in the presence of 3 μM ginkgolic acid.

Marked potentiation was observed following the application of ginkgolic acid in the nanomolar range of concentrations. As illustrated in **Figure 6E** the time-course and amplitude of I_gly_ potentiation induced by 100 nM ginkgolic acid were similar to those for α1 GlyR.

All together, these observations demonstrate that after mutation of three residues (T59A/A261G/A303S) in the α2 subunit the effect of ginkgolic acid on the receptor became similar to that observed on α1 GlyR.

## Discussion

In this study we have shown that GlyRs are modulated by ginkgolic acid in a subunit-specific manner. After pre-application of ginkgolic acid (0.5–6 min), I_gly_ mediated by α1 GlyRs expressed in CHO and neuroblastoma cells were strongly potentiated. This effect was observed at nanomolar ginkgolic acid concentration (30–100 nM). High doses of ginkgolic acid (25 μM) caused a small inhibition of α2 GlyRs, while there was no detectible effect of ginkgolic acid on amplitudes of currents mediated by α3 GlyRs or on GABA_A_Rs composed of α1/β1/γ2 subunits. These observations indicate that low concentrations of ginkgolic acid highly specifically potentiate α1 GlyRs.

The effects of ginkgolic acid on α1 GlyRs exhibit the following features. Firstly, potentiation is accompanied by significant left-shifts of dose–response curves and a decrease in EC_50_ values, suggesting modulation of gating properties of α1 GlyR channels. Analysis of dose–response curves demonstrated that 25 and 3 μM of ginkgolic acid caused a similar shift in EC_50_: respectively, from 36 to 22 μM and form 36 to 17 μM. This allows to suggest that the maximal potentiation of α1 GlyR can be achieved even at relatively low doses of the acid.

Secondly, potentiation develops slowly, on a time scale of minutes, and the strength of the effect depends on the concentration of ginkgolic acid. During application of 30 nM ginkgolic acid the onset of potentiation was observed only after 2–3 min, while high concentrations (25 μM) caused potentiation by more than 150% after only 30 s of ginkgolic acid presence in the external solution.

Recovery of glycine-evoked currents after potentiation of α1 GlyRs by ginkgolic acid, developed slowly, usually not being complete, in the time range of minutes. This could occur for two main reasons. First, the slow unbinding rate of ginkgolic acid from the potentiating site of the receptor situated in a hydrophobic membrane environment. Very low concentrations of the drug could accumulate at binding sites and produce long-lasting enhancement, similar to the inhibitory effects of lipophilic blockers of GlyR (Islam and Lynch, 2012). Second, spontaneous increase in I_gly_, as it has been previously demonstrated that during long-lasting whole-cell recordings the EC_50_ of GlyRs for glycine spontaneously increases (Fucile et al., 2000). While this spontaneous enhancement of currents was clearly distinguishable from effects of ginkgolic acid (**Supplementary Figure S1**), it could contribute to the irreversible increase.

Ginkgolic acid at very high doses (>10 μM) caused a weak inhibition of whole-cell currents mediated by receptors formed of α2 subunits, without modulating the function of α3 GlyRs and GABA_A_R. Moreover, at 1 μM ginkgolic acid was not able to modulate α2 GlyRs, confirming its selectivity to α1 GlyRs.

In order to further investigate this subunit-specific effect of ginkgolic acid we have focused on possible interaction sites for this compound inside different GlyR domains. In previous studies it has been shown that most of the residues that are responsible for GlyR modulation by ions, cannabinoids, alcohols, and anesthetics are located in the extracellular domain, in the TM2 and TM3 domains (Mihic et al., 1997; Lynch et al., 1998; Maksay et al., 2009; Yevenes and Zeilhofer, 2011b).

It has been demonstrated that extracellularly localized amino acid 52 of the α subunit is responsible for the differences in the ethanol sensitivity of GlyRs composed of homomeric α1 and α2 subunits (Mascia et al., 1996). Specifically, α1 GlyRs were more sensitive to the action of the ethanol than were α2 GlyRs or the mutant α1 (A52S) receptors. Situated in TM3 domain, residue S296 was found to be crucial for GlyR potentiation by THC, the major psychoactive component of marijuana (Xiong et al., 2011). THC more effectively potentiated currents mediated by GlyR α1 subunits than the currents mediated by α2 subunits. Mutants of α1 subunits in which serine 296 was substituted for alanine showed a decrease in the potentiation magnitude (Xiong et al., 2012).

Subunit-specific modulation of GlyRs has also been demonstrated for the endocannabinoid *N*-arachidonoyl-glycine and synthetic CB1 and/or CB2 receptor ligands (HU-210, WIN 55,212-2), which potentiate α1 GlyR and inhibit α2 GlyR (Yang et al., 2008). Searching for sites involved in positive modulation of GlyR by endocannabinoids it was revealed that substitution of three amino acids in α2 subunits for corresponding amino acids from α1 subunits T59A/A261G/A303S can convert the inhibitory effect of NA-Gly into potentiation (Yevenes and Zeilhofer, 2011a). Based on the similarity of NA-Gly and ginkgolic acid in the GlyR modulation profile we have suggested that the same amino acid residues could be responsible for α1 GlyR potentiation by ginkgolic acid.

Indeed, application of ginkgolic acid to cells expressing α2 T59A/A261G/A303S subunits resulted in (i) an increase in responses to low concentrations of glycine; (ii) a slow development of the effect, similarly as for α1 GlyR. This augmentation effect was observed at as low as 100 nM of ginkgolic acid. Our results reinforce the important role of these amino acids for specific modulation of α1 GlyR. The molecular mechanisms underlying the interaction of drugs with these residues and processes determining specificity of their action needs further analysis.

In contrast to selective potentiation of alpha 1 GlyR, ginkgolic acid caused similar acceleration of desensitization of all GlyR subunits (see, for instance, **Figures 3** and **4**). In line with previous observations (Breitinger et al., 2002) it suggests that regulation of ion channel activation and desensitization can involve different domains. The molecular determinants of desensitization may involve extracellular and TMs or interface between them (Bouzat et al., 2008; Wang and Lynch, 2011), as well as TM1–TM2 (Breitinger et al., 2001) and TM2–TM3 (Nikolic et al., 1998; Breitinger et al., 2002; Meiselbach et al., 2014) cytoplasmic domains. A recent study presented compelling experimental and modeling analysis of this phenomenon demonstrating that the internal end of TM3 and TM1–TM2 linker control desensitization (Gielen et al., 2015). As these parts of molecular sequences are identical for all GlyR subunits, in a view of the study by Gielen et al. (2015), one can suggest that regulation of desensitization by ginkgolic acid may be developed at this level.

Being lipophilic ginkgolic acid can penetrate plasma membrane and interact with various intracellular targets (Fukuda et al., 2009; Lu et al., 2012; Ma et al., 2015) causing regulation of receptor functioning through the intracellular pathways. Thus, activation of protein phosphatase 2C (PP2C) by ginkgolic acid and, consequently, stimulation of neuronal death in cell cultures has been previously demonstrated (Ahlemeyer et al., 2001). However, ginkgolic acid caused effects on PP2C at very high concentrations (>100 μM) (Ahlemeyer et al., 2001), while the effects in our experiments effects were observed at 100 nM, i.e. 1000x less concentration.

We also performed testing of ginkgolic acid action on outside-out patches from cells expressing α1 GlyRs. This configuration should accelerate washing out of intracellular components and eliminate potentiation. However, modulation was very similar to that seen during whole-cell recordings. In addition, ginkgolic acid potentiated mutant α2 GlyRs suggesting its interaction with receptor proteins.

Although these observations reduce the assumption of regulation through the intracellular pathways, this possibility is not excluded. Careful analysis in a separate study using the inside-out configuration and other approaches is necessary to clarify this question.

Several previous studies have demonstrated that cannabinoids and endocannabinois cause modulation of GyR function (Lozovaya et al., 2011). The most effective is a natural component of marijuana, THC, which at nanomolar concentrations (beginning from 30 nM) caused potentiation of α1 and α3 GlyR subunits with weak augmentation of α2 GlyR-mediated currents (Xiong et al., 2011). However, effects of other compounds from this family are complicated, as they cause direct modulation of voltage-gated and receptor-operated ion channels (see reviews, Oz, 2006). While the action of gingkolic acid on other receptors, ion channels, and synaptic networks needs future analysis, the observations presented here suggest that this compound acts as a specific enhancer of α1 GlyR subunits, with the threshold of potentiation in the range of 30 nM.

A large variety of evidence indicates that GlyR subtypes are differentially distributed in the nervous system. GlyR functions depend on subunit composition, subsynaptic localization, and activation mode, they are involved in the control of many motor and sensory pathways, including those necessary for audition, vision, respiration and nociception (Kirsch, 2006; Harvey et al., 2009; Dutertre et al., 2012; Zeilhofer et al., 2012). Thus, α1 GlyRs are primary localized in adult spinal cord, being responsible for movement and muscle tone control (Kneussel and Betz, 2000; Lynch, 2004); α2 GlyRs are important for embryonic brain development (Kneussel and Betz, 2000) and visual perception (Haverkamp et al., 2004). These receptors are dominantly expressed in prenatal brain, but their number dramatically decreases between birth and the third postnatal week (Sato et al., 1992). At the same period, the level of α1 GlyRs increases and they become widely distributed in spinal cord, retina, and brainstem nuclei (Malosio et al., 1991; Sato et al., 1992; Greferath et al., 1994; Zeilhofer et al., 2012). GlyRs are differentially expressed in hippocampus and their subcellular localization and subunit composition change over development (Aroeira et al., 2011). Thus, our observations on subunit-specific modulation of GlyRs by ginkgolic acid might be relevant for specific regulation of the physiological functions mediated by GlyRs in pathological conditions.

## Conflict of Interest Statement

The authors declare that the research was conducted in the absence of any commercial or financial relationships that could be construed as a potential conflict of interest.

## Supplementary Material

**Figure S1 |Differences in the time course of elevation of current amplitudes induced by glycine (30 μM) at spontaneous run up and during application of 100 nM ginkgolic acid.**

## ABBREVIATIONS

CB receptor: cannabinoid receptor
cDNA: complementary deoxyribonucleic acid
CHO cells: Chinese hamster ovary cells
DMSO: dimethyl sulfoxide
EC_50_: half-maximal concentration
GABA: γ-aminobutyric acid
GABA_A_R: γ-aminobutyric acid receptor, subtype A
GlyR: glycine receptor
I_gly_: glycine-induced current
THC: Δ^9^-tetrahydrocannabiol
TM: transmembrane domain
WT: wild type.
