# Supplementary figures and images for: Selective potentiation of alpha 1 glycine receptors by ginkgolic acid
Source: Front Mol Neurosci. 2015 Oct 29;8:64. doi: 10.3389/fnmol.2015.00064 (PMC4624854; doi:10.3389/fnmol.2015.00064)

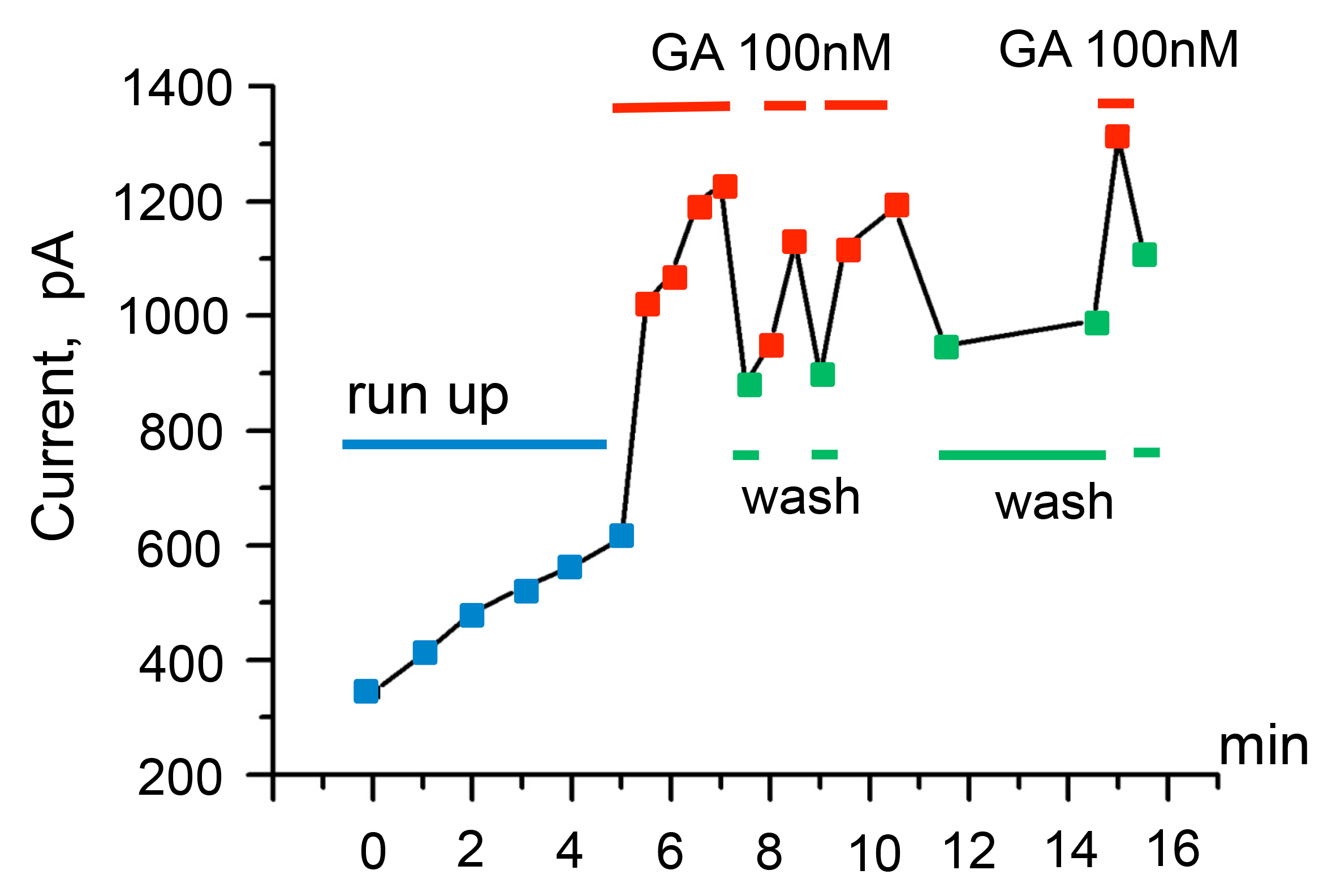

Supplement: Supplementary file 1 [file Image_1.JPEG]
